# The complete mitochondrial genome of the widespread freshwater limpet *Ferrissia californica*

**DOI:** 10.1080/23802359.2025.2487068

**Published:** 2025-04-15

**Authors:** Nino Kachlishvili, Clément Schneider, Ani Bikashvili, Barbara Feldmeyer, Markus Pfenninger, Levan Mumladze

**Affiliations:** ^a^ Institute of Zoology, Ilia State University, Tbilisi, Georgia; ^b^ Abteilung Bodenzoologie, Senckenberg Gesellschaft für Naturforschung, Görlitz, Germany; ^c^ Molecular Ecology Group, Senckenberg Biodiversity and Climate Research Centre, Frankfurt, Germany

**Keywords:** Cryptic invader, genome construction, phylogeny, planorbidae

## Abstract

*Ferrissia californica* (Rowell, 1863) (Gastropoda: Hygrophila: Planorbidae) is a globally distributed freshwater limpet native to North America. Based on the specimens collected in Georgia, we aimed to sequence and annotate the mitochondrial genome of *F. californica* for the first time. The mt-genome spans 13526 bp containing 13 protein-coding, 2 ribosomal RNA, and 22 transfer RNA genes. Comparisons with the mitochondrial genomes of other gastropod molluscs revealed differences in gene organization. A phylogenetic reconstruction based on 12 protein-coding genes of several representatives from the Planorbidae and Limnaeidae families placed *F. californica* and *Laevapex fuscus* (C. B. Adams, 1840) as sister taxa.

## Introduction

**Figure 1.**
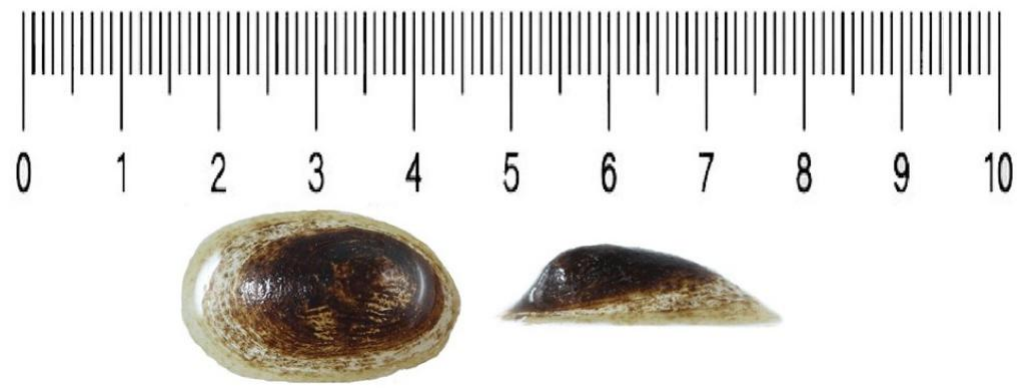
Shell of *Ferrissia californica*. The photo was taken at Ilia State University. Photograph by Nino Kachlishvili and Armen Seropian. Voucher number Fe2-e41d4p2. The length of the shell is 2.8 mm; the width is 1.8 mm and the height is 1 mm. Shell has a blunted apex which is slightly shifted to the right.

The North American freshwater limpet *Ferrissia californica* (Rowell, 1863) (Gastropoda: Planorbidae), was first described in 1863. During the 1863–1920 years in North America, *F. californica* (*sensu stricto*), was represented under different binomens (Bash 1963). In the 1940s, species of *Ferrisia* were found in Europe (Walther et al. 2006; Tokinova 2012), and since then they have been recorded in European and Asian countries (Walther et al. 2006; Son 2007). Morphological analyses and the results of molecular-genetic studies, which included mostly partial mitochondrial gene analyses like CO1 and ribosomal subunits, indicate that *F. californica* is the only representative of the genus in Eurasia, and is a cryptic multicontinental invader (Walther et al. 2006; Beran and Horsák 2007; Son 2007; Marrone et al. 2011; Raposeiro et al. 2011; Marrone et al. 2014; Eduardo et al. 2015; Vinarski and Palatov 2018). *F. californica* has a small, fragile shell (Figure 1) and can be found in different types of aquatic habitats (Bash 1963; Semenchenko and Laenko 2008). Its record in the South Caucasus (as well as in the subterranean environment) was first evidenced in 2016 in the Iasoni cave near the city of Kutaisi (western Georgia) (Vinarski and Palatov 2018). Thereafter, the species has been recorded repeatedly in western Georgia (our unpublished data), indicating the rapid range expansion locally. Given the absence of reference genomes, this manuscript aims to present the first full mitochondrial genome of *F. californica* and characterize its structure and nucleotide composition.

## Materials and methods

The sample of *F. californica* was collected in the Samegrelo region; river Sadagalis Tskali (N42.315836, E41.983868) on August 10, 2019. We collected samples by hand on the stony, shallow stream. The sample was preserved in 96% ethanol and stored at −20 °C in Ilia State University, Institute of Zoology (https://research.iliauni.edu.ge/en/institution/24-zoologiis-instituti Levan Mumladze lmumladze@gmail.com) voucher Fe2-e41d4p2. Species were identified by morphological features, like size and form of the shell, and apex characteristics (Piechocki and Wawrzyniak-Wydrowska 2016) and with BLAST by comparing sequencing results with sequences in NCBI database (Johnson et al. 2008).

DNA was extracted from the whole body of the specimen with the QIAGEN blood and tissues plus kit (Qiagen, Germany). Library preparation and genome sequencing were conducted at Novogene company, with sequencing platform - Illumina Sequencing PE150, Library type Plant and animal whole genome library preparation (350 bp) and data requirement 10 G of raw data per sample. Adapter trimming was done using Trimmomatics 0.39 (Bolger et al. 2014), and non-invertebrate metazoan reads were discovered and removed using Kraken2 2.0.8. (Wood et al. 2019). The mitochondrial genome was assembled using GetOrganelle pipeline v 1.7.6.1 (Jin et al. 2020) with SPADes v 3.15.0 (Prjibelski et al. 2020). The sequencing library result of 64,939,428 paired 150 bp reads were mapped on the reference genome of North American freshwater limpet *Laevapex fuscus* (C.B. Adams, 1841) (Genbank Accession Number MN830918) Assembly quality was checked using the Galaxy server (Community 2024) and the mapping to the reference was performed with BWA V0.7.18-r1243 (Li and Durbin 2010). The mapping quality was confirmed using QualiMap v.2.3 (Okonechnikov et al. 2016) (coverage plot is shown in Figure S1). The mitochondrial genome of *Ferrissia* was automatically annotated by MITOS2 (Bernt et al. 2013), with Infernal v 1.1 (Nawrocki and Eddy 2013) and Geneious Prime 2022.1.1. (Biomatters, New Zealand). The final annotation was compiled manually from a combination of both. Expasy (Gasteiger et al. 2003), alignment with genomes that matched a higher percentage with *F. californica*, and BlastX (Johnson et al. 2008) were the main helpers in protein-coding gene position clarification, and determination of start and stop codons. The locations of transfer RNAs were checked with Arwen v1.2. (Laslett and Canbäck 2008) and MITOS2. Protein coding and tRNA genes positions were manually corrected in Geneious Prime.

The Phylogenetic reconstruction using 12 protein-coding genes was performed including *F. californica* PP473661 and most closely related taxa of Planorbidae and Limnaeidae family for which mitogenomes are available (Figure 3). The *atp8* gene was excluded due to its absence in genomes of interest. The saturation test was done by DAMBE ver7.3.12 (Xia 2018). Protein coding gene alignment and genetic distance counting was performed by MEGA-X (Kumar et al. 2018) The best-fitting evolutionary model was tested using Jmodeltest 2.1.10 (Posada 2008) and the phylogenetic tree was built using RAxMLGUI 2.0.13 (Edler et al. 2021) with 1000 bootstrap replicates.

## Results

**Figure 2.**
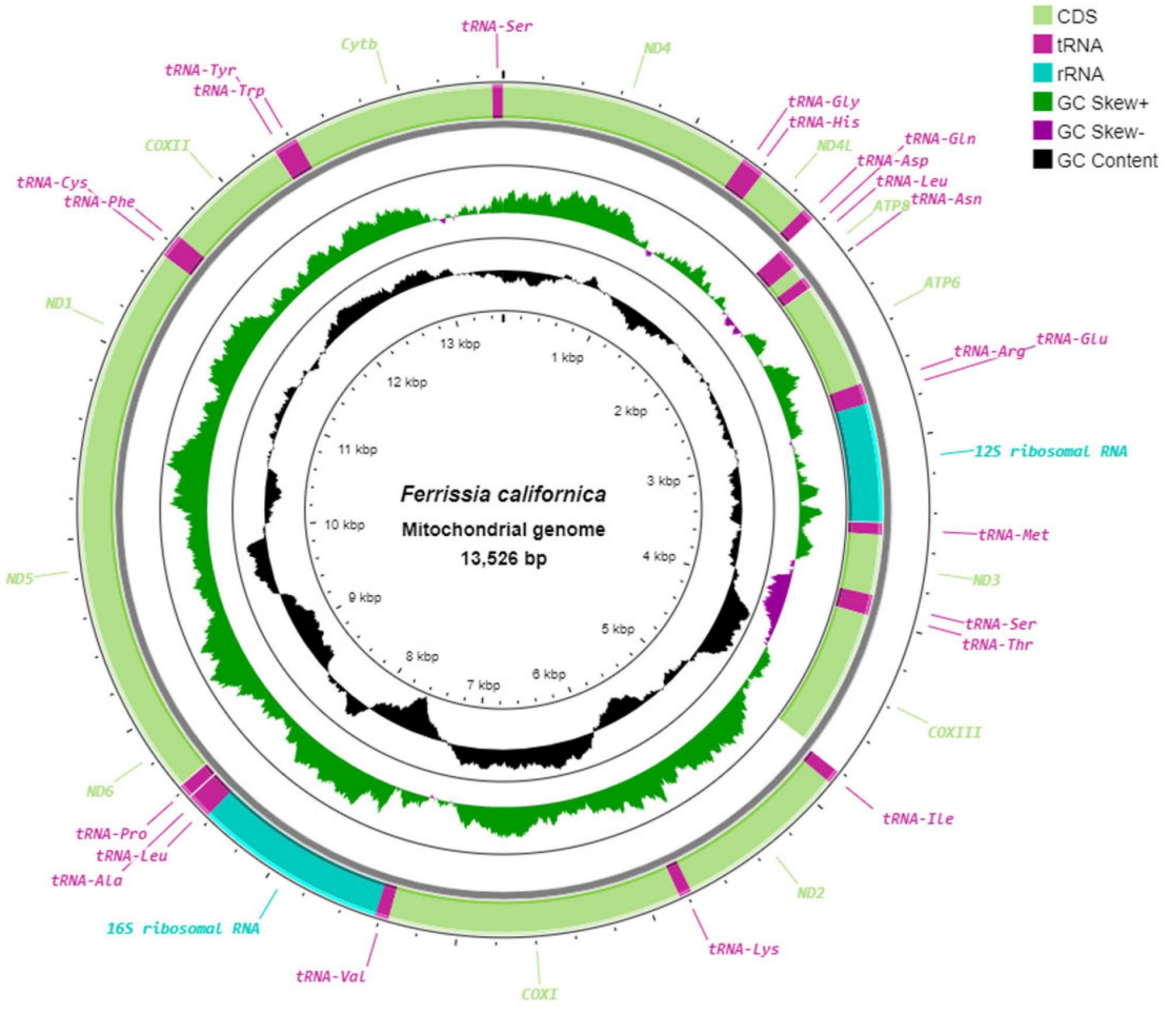
The mitochondrial genome of *Ferrissia californica* PP473661. The genome map was visualized with Proksee (Grant et al. 2023). Genes inside of the circle are encoded on the reverse strand, and genes outside of the circle are encoded on the forward strand.

**Figure 3.**
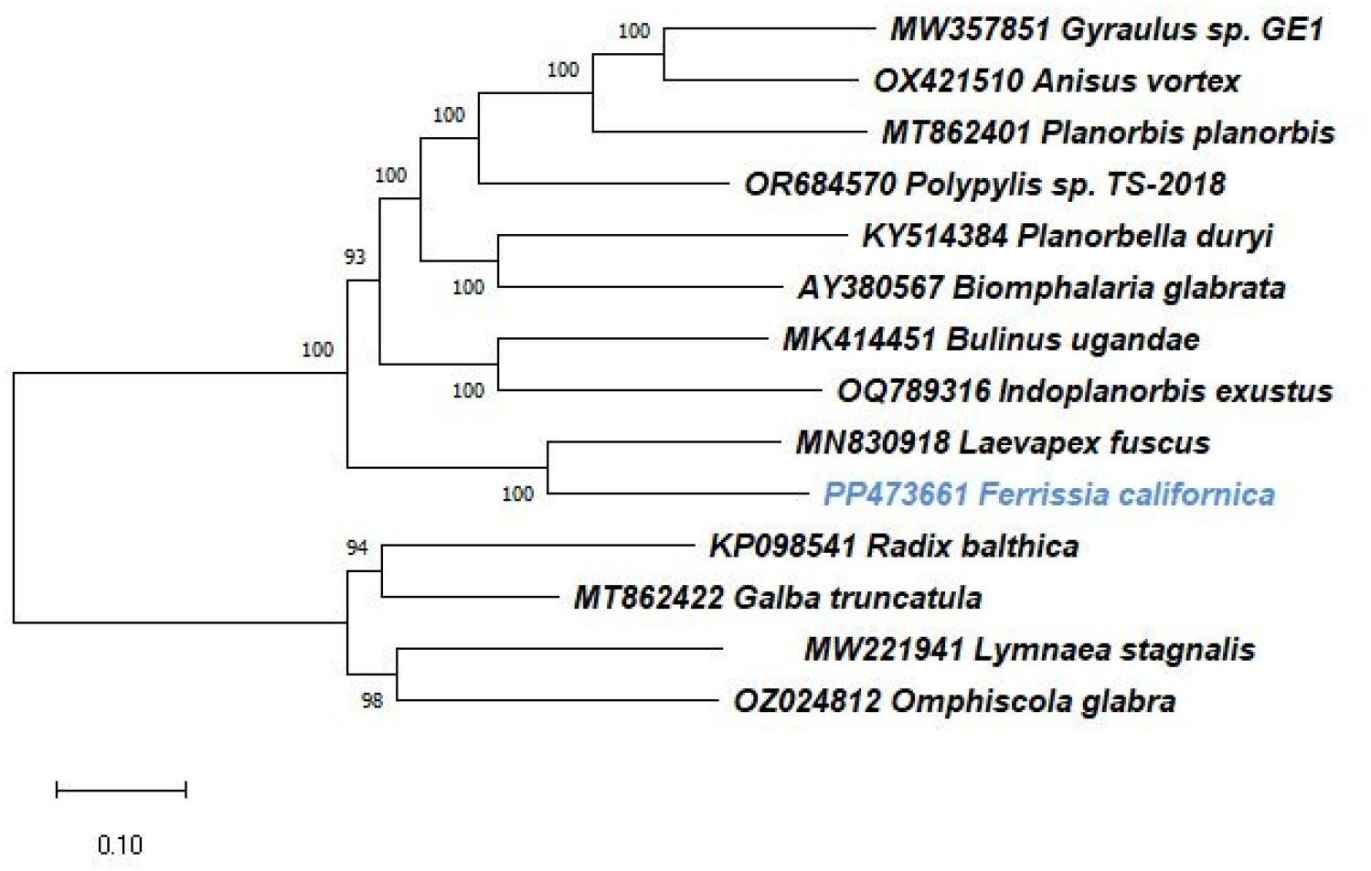
The reconstructed phylogenetic relationship of *Ferrissia californica* (Rowell 1863) PP473661 with most closely related species from Planorbidae and Limnaeidae family: *Biomphalaria glabrata* (Say 1818) AY380567 (DeJong, Aidan, and Ademat 2016); *Planorbella duryi* (Wetherby, 1879) KY514384 (Schultz et al. 2018); *Gyraulus* sp. Charpentier, 1837 MW357851 (unpublished); *Laevapex fuscus* (C. B. Adams, 1840) MN830918 (unpublished); *Bulinus ugandae* Mandahl-Barth, 1954 MK414451 (Zhang et al. 2022); *Indoplanorbis exustus* (Deshayes 1833) OQ789316 (unpublished); *Anisus vortex* (Linneaus, 1758) OX421510 (Skipp and Ablett 2023); *Planorbis planorbis* (Linneaus 1758) MT862401 (Rempel et al. 2021); *Polypylis* sp. Pilsbry, 1906 OR684570 (Tao et al. 2024); as an outgroup: *Radix balthica* (Linnaeus, 1758) (accepted name: *Ampullaceana balthica* (Linnaeus 1758)) KP098541 (Feldmeyer et al. 2015); *Galba truncatula* (O. F. Müller 1774) MT862422 (unpublished); *Lymnaea stagnalis* (Linnaeus 1758) MW221941 (unpublished); *Omphiscola glabra* (O. F. Müller, 1774) OZ024812 (unpublished).

The mitochondrial genome of *F. californica* is a 13,526 bp long molecule (GenBank accession number PP473661) (Figure 2) with an overall base composition of 33.1% for A; 10.8% for C; 13.5% for G; 42.6% for T; and with GC content 24.3% and AT content 75.7%. It contains 13 protein-coding, 2 ribosomal RNA, and 22 transfer RNA genes. Thirteen out of 37 genes are encoded on the heavy strand of the genome, while other genes are encoded on the light strand. Protein coding genes are initiated with 5 different codons: TTA, ATA, ATT, ATC, and ATG. Almost all protein-coding genes terminate with a TAA stop codon, except for *nad5* stopping with TAG. The two ribosomal RNA genes are encoded on different strands: *rrnS* (688 bp) on the heavy strand and *rrnL* (1011 bp) on the light strand. The comparison of *F. californica* and the reference genomes of *L. fuscus* with other gastropods shows that the location of most of the genes coincides with each other, they also have slight changes in gene organization *nad4l*, *cox2*, and *nad4* and also *trnC* and *trnF*; *trnW* and *trnY* and *trnS* can be found on a different position (Figure S2). The saturation test showed no saturation (observed Iss 0.5702 vs. critical Iss.c 0.8293). The dataset resulted in 20% to almost 40% divergence between the species, and 32%, particularly between *F. californica* and *L. fuscus*. The result of ML phylogeny based on GTR+G + I model is shown in Figure 3.

## Discussion and conclusions

The phylogenetic analyses based on the protein-coding genes of *F. californica* and other Planorbidae and Limnaeidae species indicate that *F. californica* with *L. fuscus* are clustered within the Planorbidae species presented in this study (Figure 3). Compared to several other species they present different gene positions. In particular, *cox2* is positioned before *cytb* while *nad4* has moved after *cytb*, followed by *nad4l*. Changes are observed in the transfer RNA positions: *trnC* and *trnF* are moved forward with *cox2*, and *trnW* and *trnY* are shifted in front of *cytb*. One of the *trnS* has moved forward with *nad4* (Figure S2). An interesting outcome is observed in gene nucleotide composition, TTA initiating codon, which is the *cox1* start codon in *F. californica* differs from other most closely related mitochondrial genomes from genbank. Despite this difference, according to the analyses mentioned in materials and methods, TTA codon was kept as the start codon for *cox1* gene. Since molluscs mitochondrial genes can have different start and stop codons (Ghiselli et al. 2021), for *F.californica* TTA might be an initiative codon for *cox1* gene. In NCBI database *cox1* was marked as incomplete. Despite these structural changes in the mitochondrial genome, which are rather frequent in the molluscan evolution (Ghiselli et al. 2021), the basic structure of the genome, seen in many types of molluscs, is unchanged (Nolan et al. 2014; Schultz et al. 2018; Zhang et al. 2022, 2018).

## Supplementary Material

Supplementary material.docx

## Data Availability

The genome sequence data is available in GenBank of NCBI at [https://www.ncbi.nlm.nih.gov] under the accession number PP473661. The associated BioProject, SRA, Bio-sample numbers are PRJNA1116555, SRR29182321, and SAMN41534510.
