# Supplementary material for: The complete mitochondrial genome of the widespread freshwater limpet Ferrissia californica
Source: Mitochondrial DNA B Resour. 2025 Apr 15;10(5):382–6. doi: 10.1080/23802359.2025.2487068 (PMC12001849; doi:10.1080/23802359.2025.2487068)
Supplement: Supplementary material.docx [file TMDN_A_2487068_SM9364.docx]

**The complete mitochondrial genome of the widespread freshwater limpet *Ferrissia californica***

Nino Kachlishvili ^a*^, Clément Schneider ^b^, Ani Bikashvili ^a^, Barbara Feldmeyer ^c^, Markus Pfenninger ^c^, Levan Mumladze ^a^

a Institute of Zoology, Ilia State University, Tbilisi, Georgia; b Abteilung Bodenzoologie, Senckenberg Gesellschaft für Naturforschung, Görlitz, Germany; c Molecular Ecology Group, Senckenberg Biodiversity and Climate Research Centre, Frankfurt, Germany

**Abstract**

*Ferrissia californica* (Rowell, 1863) (Gastropoda: Hygrophila: Planorbidae) is a globally distributed freshwater limpet native to North America. It can be found in rivers, streams, coastal zones of lakes, and even fountains. *F. californica* has been known in West Georgia since 2016. Based on the specimens collected in Georgia, we aimed to sequence and annotate the mitochondrial genome of *F. californica* for the first time. The mt-genome spans 13526 bp containing 13 protein-coding, 2 ribosomal RNA, and 22 transfer RNA genes. Comparisons with the mitochondrial genomes of other gastropod molluscs revealed differences in gene organization. The genes *nad4, nad4l,* and *cox2*, as well as several transfer RNA genes, exhibit positional changes. A phylogenetic reconstruction based on 12 protein-coding genes of several representatives from the Planorbidae and Limnaeidae families placed *F. californica* and *Laevapex fuscus* (C. B. Adams, 1840) as sister taxa.

**Keywords: 1** Cryptic invader **2** Genome construction **3** phylogeny **4** Planorbidae

[***nino.kachlishvili.1@iliauni.edu.ge**](mailto:*nino.kachlishvili.1@iliauni.edu.ge)


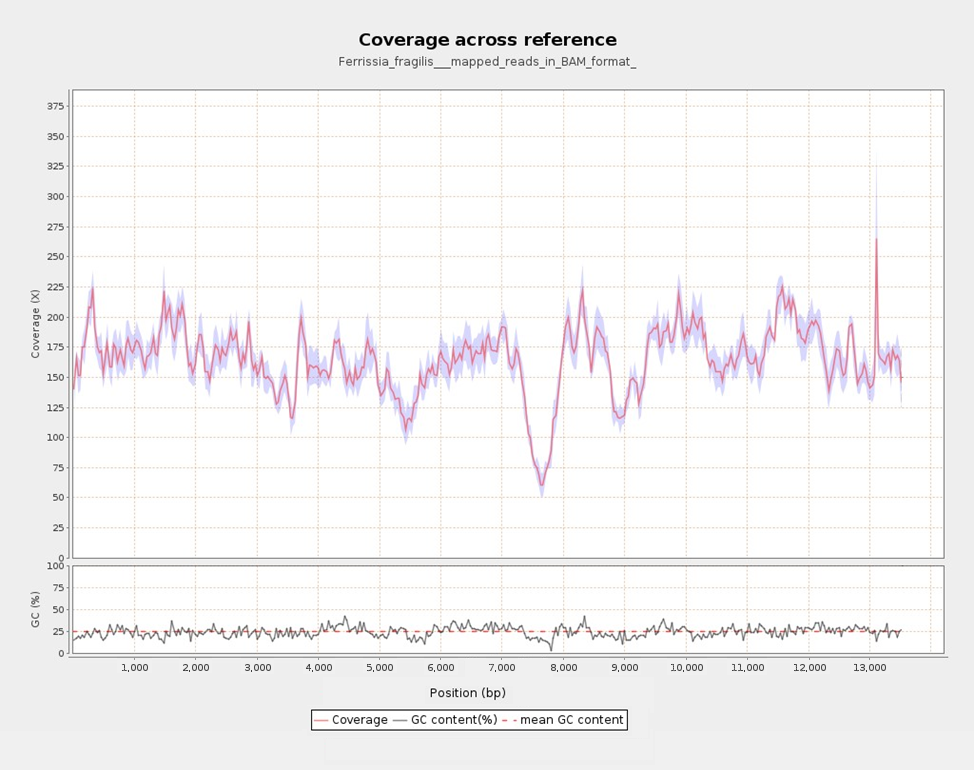


**Figure S1.** The complete mitochondrial genome of the widespread freshwater limpet *Ferrissia californica*. The coverage plot of the *F. californica* mitochondrial genome. Number of mapped bases: 223.076.6 bp; mean mapping quality: 59.83; mean coverage: 164.9243.

**
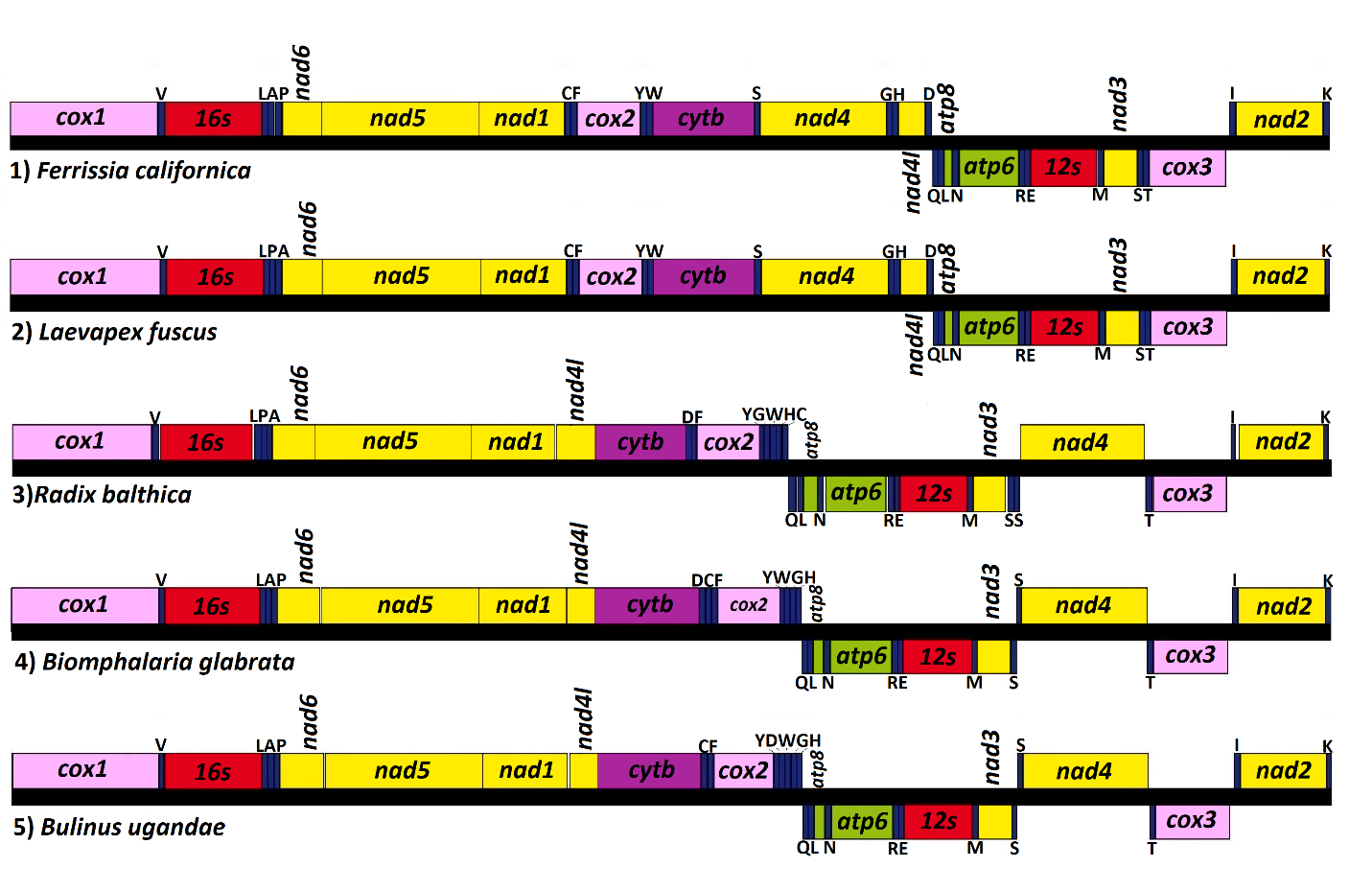
Figure S2.** Mitochondrial genome organization in *Ferrissia californica* (Rowell, 1863) PP473661 and genome comparison with other gastropods: *Laevapex fuscus* (C. B. Adams, 1840) MN830918 (unpublished), *Radix balthica* (Linnaeus,1758) (accepted as: *Ampullaceana balthica* (Linnaeus, 1758)) KP098541 (Feldmeyer et al. 2015), *Biomphalaria glabrata* (Say, 1818) AY380567 (DeJong, Aidan, and Ademat 2016), *Bulinus ugandae* Mandahl-Barth, 1954 MK414451 (Zhang et al. 2022). Circular molecules are depicted in linear bars. The *cox1*-*3* indicates cytochrome oxidase subunits (pink); *12S* and *16S* - small and large ribosomal subunits (red); *nad1-6* and *nad4L* - NADH dehydrogenase subunits (yellow); *cytb* - cytochrome B (purple); *atp8 and atp6* ATP synthase (green). Transfer RNA genes are represented by their one-letter amino acid codes (dark blue).
